# Genome-Wide Patterns of Population Structure and Linkage Disequilibrium in Farmed Nile Tilapia (*Oreochromis niloticus*)

**DOI:** 10.3389/fgene.2019.00745

**Published:** 2019-09-04

**Authors:** Grazyella M. Yoshida, Agustín Barria, Katharina Correa, Giovanna Cáceres, Ana Jedlicki, María I. Cadiz, Jean P. Lhorente, José M. Yáñez

**Affiliations:** ^1^Facultad de Ciencias Veterinarias y Pecuarias, Universidad de Chile, Santiago, Chile; ^2^Benchmark Genetics Chile, Puerto Montt, Chile; ^3^Nucleo Milenio INVASAL, Concepción, Chile

**Keywords:** effective population size, LD decay, linkage disequilibrium, *Oreochromis niloticus*, population structure

## Abstract

Nile tilapia (*Oreochromis niloticus*) is one of the most produced farmed fish in the world and represents an important source of protein for human consumption. Farmed Nile tilapia populations are increasingly based on genetically improved stocks, which have been established from admixed populations. To date, there is scarce information about the population genomics of farmed Nile tilapia, assessed by dense single nucleotide polymorphism (SNP) panels. The patterns of linkage disequilibrium (LD) may affect the success of genome-wide association studies (GWAS) and genomic selection (GS), and also provide key information about demographic history of farmed Nile tilapia populations. The objectives of this study were to provide further knowledge about the population structure and LD patterns, as well as, estimate the effective population size (*N* *_e_*) for three farmed Nile tilapia populations, one from Brazil (POP A) and two from Costa Rica (POP B and POP C). A total of 55 individuals from each population, were genotyped using a 50K SNP panel selected from a whole-genome sequencing (WGS) experiment. The first two principal components explained about 20% of the total variation and clearly differentiated between the three populations. Population genetic structure analysis showed evidence of admixture, especially for POP C. The contemporary *N* *_e_* estimated, based on LD values, ranged from 78 to 159. No differences were observed in the LD decay among populations, with a rapid decrease of *r* *^2^* with increasing inter-marker distance. Average *r* *^2^* between adjacent SNP pairs ranged from 0.19 to 0.03 for both POP A and C, and 0.20 to 0.03 f or POP B. Based on the number of independent chromosome segments in the Nile tilapia genome, at least 9.4, 7.6, and 4.6K SNPs for POP A, POP B, and POP C respectively, are required for the implementation of GS in the present farmed Nile tilapia populations.

## Introduction

Nile tilapia (*Oreochromis niloticus*) is one of most important farmed fish species worldwide (FAO, 2018). Breeding programs established since the 1990s have played a key role in improving commercially important traits and expanding Nile tilapia farming. The Genetically Improved Farmed Tilapia (GIFT) is the most widespread tilapia breeding strain (Lim and Webster, 2006), which has been introduced to several countries in Asia, Africa and Latin America (Gupta and Acosta, 2004). The genetic base of GIFT was established from eight African and Asian populations, and after six generations of selection, the genetic gains ranged from 10 to 15% per generation for growth-related traits (Eknath et al., 1993), providing evidence that selective breeding using phenotype and pedigree information can achieve high and constant genetic gains (Gjedrem and Rye, 2018).

The recent development of dense SNP panels for Nile tilapia (Joshi et al., 2018; Yáñez et al., 2019) will provide new opportunities for uncovering the genetic basis of important commercial traits; especially in those traits that are difficult or expensive to measure in selected candidates. As has been demonstrated for different traits in salmonid species, the incorporation of genomic evaluations in breeding programs is expected to increase the accuracy of breeding values, compared to pedigree-based methods (Tsai et al., 2016; Bangera et al., 2017; Correa et al., 2017; Sae-Lim et al., 2017; Yoshida et al., 2017; Barria et al., 2018b; Vallejo et al., 2018; Yoshida et al., 2019a).

Genomic studies exploit the linkage disequilibrium (LD) between SNPs and quantitative trait locus (QTL) or causative mutation. Thus, knowing the extent and decay of LD within a population is important to determine the number of markers that are required for successful association mapping and genomic prediction (de Roos et al., 2008; Khatkar et al., 2008; Porto-Neto et al., 2014; Brito et al., 2015). Therefore, when low LD levels are present within a population, a higher marker density is required to capture the genetic variation across the genome (Khatkar et al., 2008). In addition, LD patterns provide relevant information about past demographic events including response to both natural and artificial selection (Slatkin, 2008). Therefore, the LD estimates throughout the genome, reflects the population history and provides insight about the breeding system and patterns of geographic subdivision, which can be explored to study the degree of diversity in different populations.

To date, the most widely used measures of LD between two loci are Pearson’s squared correlation coefficient (r^2^) and Lewontin’s D’ (commonly named D’). Values lower than 1 for D’ indicate loci separation due to recombination, while D’ = 1 indicates complete LD between loci, i.e. no recombination. However, this parameter is highly influenced by allele frequency and sample size. Thus, high D’ estimations are possible even when loci are in linkage equilibrium (Ardlie et al., 2002). Therefore, LD measured as r^2^ between two loci is suggested as the most suitable measurement for SNP data (Pritchard and Przeworski, 2001).

LD patterns have been widely studied in different livestock species, such as sheep (Prieur et al., 2017), goats (Mdladla et al., 2016), pigs (Ai et al., 2013), beef (Espigolan et al., 2013; Porto-Neto et al., 2014) and dairy cattle (Bohmanova et al., 2010). In aquaculture, recent studies have aimed at characterizing the extent and decay of LD in farmed species, such as Pacific white shrimp (Jones et al., 2017), Pacific oyster (Zhong et al., 2017), rainbow trout (Rexroad and Vallejo, 2009; Vallejo et al., 2018), coho salmon (Barria et al., 2018a) and Atlantic salmon (Hayes et al., 2006; Gutierrez et al., 2015; Kijas et al., 2016; Barria et al., 2018c). However, to date there is scarce information about population genomic structure and LD in farmed Nile tilapia assessed by the use of dense SNP panels. The assessment of LD patterns in Nile tilapia is still limited to a few studies in which either a small number of markers (14 microsatellites) (Sukmanomon et al., 2012) and individuals (4 to 23 samples) (Hong Xia et al., 2015) have been used. Recently, the construction of a dense linkage map for Nile tilapia suggested a sigmoid recombination profile in most linkage groups (LG), showing higher recombination rates in the middle and lower recombination at the end of the LGs (Joshi et al., 2018). These patterns are consistent with the high LD levels found in the end of almost all chromosomes in a hybrid Nile tilapia population (Conte et al., 2019). The objectives of the present study were to i) estimate the population structure and genetic differentiation; ii) to assess the genome-wide levels of LD and iii) determine the effective population size among three Nile tilapia breeding populations established in Latin America.

## Methods

### Populations

Samples were obtained from three different commercial breeding populations established in Latin America, originated from admixed stocks imported from Asia and genetically improved for growth rate for more than 20 generations. Individuals from population A (POP A) belong to the AquaAmerica (Brazil) breeding population, where the animals are evaluated in cage-based production systems and have been artificially selected during three generations for improved growth rate using daily weight gain as selection criteria. This population was imported from GIFT Malaysia in 2005 for breeding and farming purposes. Individuals from population B (POP B) and C (POP C) were obtained from Aquacorporación Internacional (Costa Rica) and correspond to fish from the seventh and eighth generation, respectively, of selection for improved growth-related traits (body weight at 400 g as selection criteria) under pond system production. The POP B breeding population is a mixture of the GIFT strain (8^th^ generation), POP C and the wild strains from Egypt and Kenya used to generate the GIFT strain. The POP C breeding population represents a combination of genetic material from Israel, Singapore, Taiwan and Thailand. Therefore, the three breeding populations are considered recently admixed populations; which are directly or indirectly related to the GIFT strain. Based on the overall identical by descent (IBD) alleles, average relatedness between individuals, within each population, was estimated using Plink v1.90 (Purcell et al., 2007), through the --genome option.

### Genotyping

The genotypes were selected from a whole-genome sequencing experiment aimed at designing a 50K SNP Illumina BeadChip, which is described in detail by Yáñez et al. (2019). Briefly, caudal fin-clip were sampled from 59, 126 and 141 individuals belonging to POP A, POP B and POP C, respectively. Genomic DNA was purified from all the samples using the DNeasy Blood & Tissue Kit (QIAGEN) according to the manufacturer’s protocol (http://www.bea.ki.se/documents/EN-DNeasy%20handbook.pdf). Whole-genome sequencing was performed using multiplexing of four bar-coded samples per lane of 100bp paired-end in the Illumina HiSeq 2500 machine. The sequences were trimmed and aligned against the genome assembly O_niloticus_UMD_NMBU (Conte et al., 2019). About 36 million polymorphic sites were discovered after variant calling using the Genome Analysis Toolkit GATK (McKenna et al., 2010). A list of 50K SNP were selected based on quality of genotype and site, number of missing values, minor allele frequency (MAF), unique position in the genome, and even distribution across the genome as described by Yáñez et al. (2019). Genotype quality control (QC) was performed within each population separately, excluding SNPs with MAF lower than 5%, Hardy–Weinberg Equilibrium P-value < 1e^−06^, and missing genotypes higher than 70%. Animals with a genotype call rate below 95% were discarded. Subsequent analyses were done using the common markers along the three populations after QC (**Table 1**). Using the --genome function from Plink, animals from POP B and POP C with the highest identical by descent (IBD) were excluded (Gutierrez et al., 2015), to use a similar sample size among populations.

**Table 1:** Summary of results from quality control of SNPs for each farmed Nile tilapia population.

Parameters	Populations		
	POP A	POP B	POP C
Minor allele frequency	9,823	2,779	2,478
Hardy–Weinberg equilibrium	163	13	32
Call-rate	105	1	1
Removed*	3,007	10,305	10,587
Final number of SNPs**	33,236	33,236	33,236

*To selected common markers among populations.

**From a total of 46,334 SNPs.

### Population Structure

We investigated population differentiation calculating the pairwise Weir and Cockerham’s F_st_ (Weir and Cockerham, 1984) estimator across all loci among populations, using VCFTools (Danecek et al., 2011) software. We used the software Plink v1.09 (Purcell et al., 2007) to calculate observed (Ho) and expected (He) heterozygosity of samples for each of the three populations and for genetic differentiation through principal component analysis (PCA). The results of the first two PCAs were plotted along two axes using R scripts (R Core Team, 2016). Additionally, the population structure was examined using a hierarchical Bayesian model implemented in STRUCTURE software v.2.3.4 (Pritchard et al., 2000). We used three replicates of K value ranging from 1 to 12, a burn-in of 20,000 iterations and running of 50,000. To choose the best K value we computed the posterior probability of each K as suggested by Pritchard et al. (2000).

### Estimation of Linkage Disequilibrium and Effective Population Size

We used the Pearson’s squared correlation coefficient (r^2^) to estimate the LD between each pair of markers. We used Plink v1.09 using the parameters --ld-window-kb 10000, --ld-window 99999, and --ld-window-r2 set to zero to calculate the LD between all pairs of SNPs on each chromosome. Based on the physical distance of each SNP pair, we created bins of 100 kb among all pairwise combinations. The extent and decay of the LD, for each population, were visualized by plotting the average r^2^ within each bin, spanning a physical distance from 0 to 10 Mb. We used the software SNeP v1.1 (Barbato et al., 2015) to estimate the historical effective population size (*N*_e_). Considering the LD within each population, *N* *_e_* was estimated using the following equation proposed by Corbin et al. (2012):

$${\text{N}}_{\text{et}}=\frac{1}{\left(4f({\text{c}}_{\text{t}})\right)}\left(\frac{1}{\text{E}\left[r_{\mathrm{adj}}^{2}|{\text{c}}_{\text{t}}\right]}-\alpha \right)$$

where *N* *_et_* is the effective population size *t* generations ago, the expectation (E) of $r_{\mathrm{adj}}^{2}$ is the estimated LD corrected for sample size $\left(\;{\text{r}}_{\text{adj}}^{2}={\text{r}}^{2}-1/\text{sample size}\right)$ and is conditional to the markers being the appropriate distant apart given *t* and mapping function *f*(c_t_), and α is the adjustment for mutation rate (α = 2, indicate the presence of mutation). Values for number and size of each bin were used as default (30 and 50 Kb, respectively). Based on the relatively small number of SNP per chromosome, *N*_e_ per chromosome was calculated using harmonic mean (Alvarenga et al., 2018). Using the LD method, we calculated the contemporary population size using the software NeEstimator v2.01 (Do et al., 2014), with a non-random mating model and a critical value of 0.05. Additionally we fitted a linear regression model for historical values of *N* *_e_* to calculate the contemporary *N* *_e_*.

Estimation of the effective number of chromosome segments (M_e_) was assessed based on the following formula proposed by Daetwyler et al. (2010):

$${\text{M}}_{\text{e}}=4N_{e}\text{L}$$

where *N* *_e_* is the effective population size and L is the length of the Nile tilapia genome in Morgans.

## Results

### Quality Control

**Figure 1:**
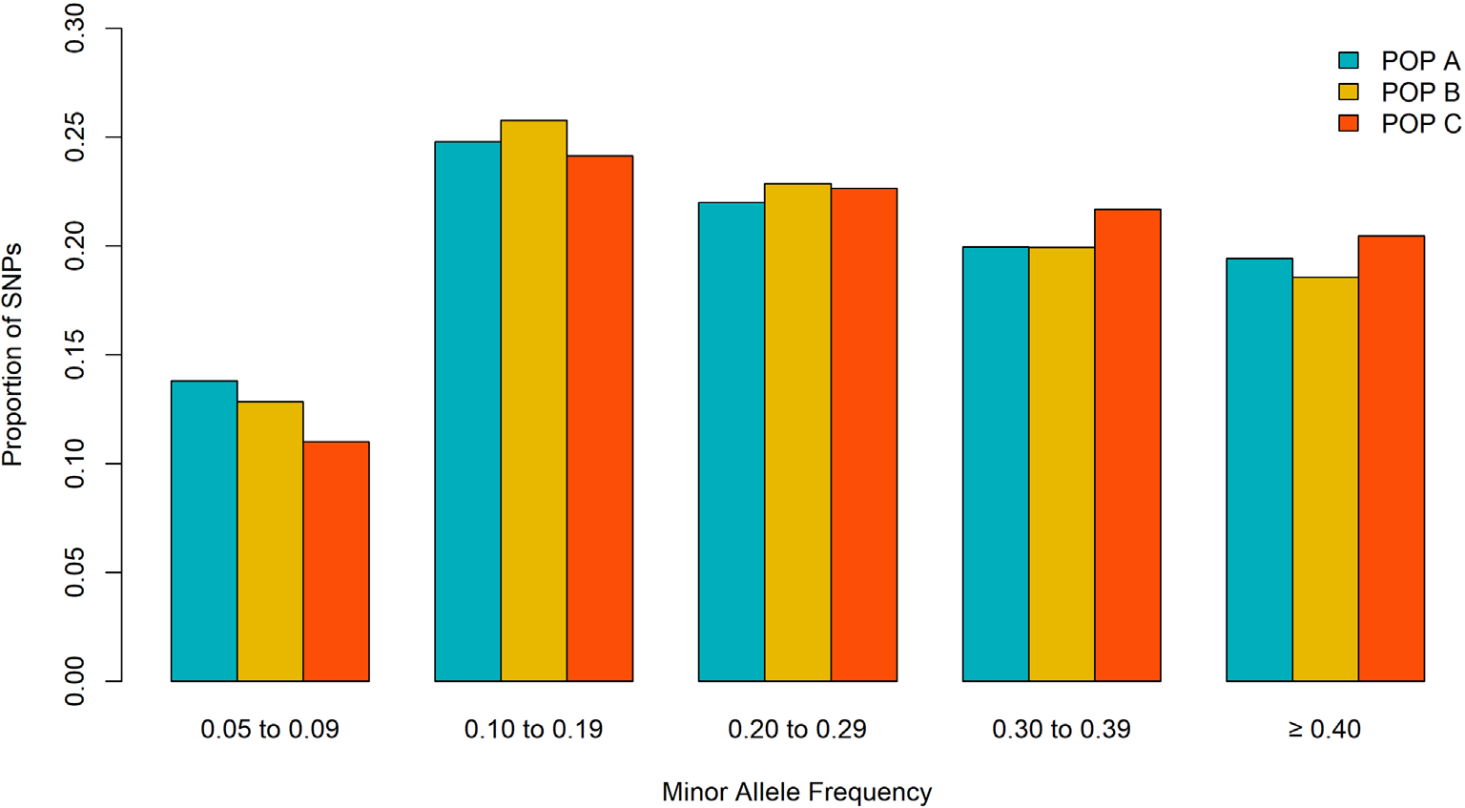
Proportion of SNPs for different minor allele frequency for three Nile tilapia population.

Out of the initial 46,334 markers, a total of 33,236 markers were shared among the three populations after QC criteria. The MAF < 0.05 excluded the higher number of SNPs along populations (ranging from ∼ 3K to ∼ 9 K) (**Table 1**). After QC, all three populations showed a similar mean MAF value of 0.26 ± 0.13 and similar proportion of SNPs for each MAF class (**Figure 1**). The lower (∼ 0.13) and higher (∼ 0.25) proportion of SNP were observed in the MAF classes ranging from 0.05 to 0.09 and 0.10 to 0.19, respectively.

For downstream analysis, we selected 55 animals for each population based on identity by descent analysis (IBD). We discarded a total of 4, 71 and 86 animals from POP A, POP B and POP C, respectively. Thus, the average relatedness within populations was 0.00 ± 0.01.

### Population Structure

**Figure 2:**
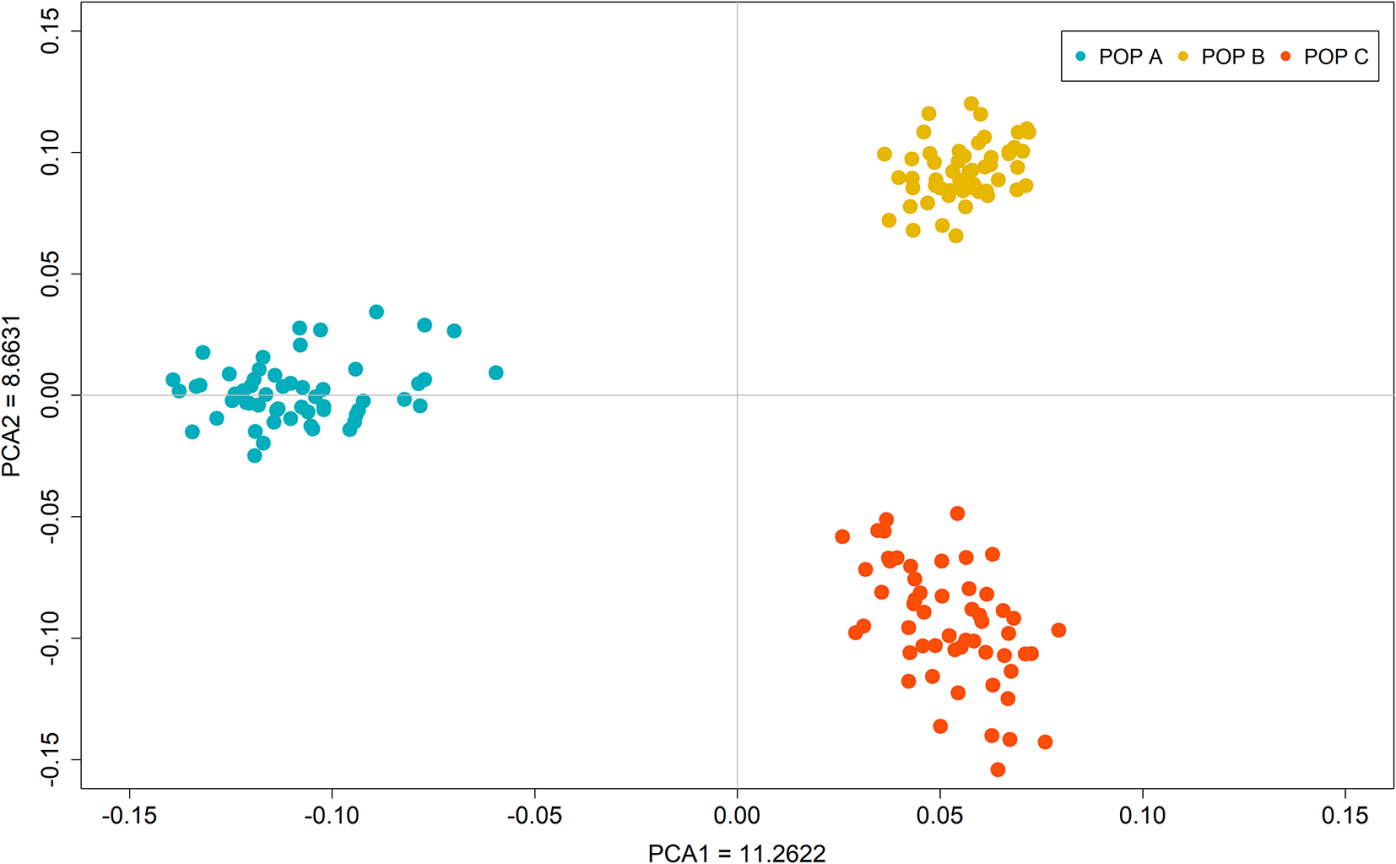
Principal component analysis of the autosomal genotypic data of three Nile tilapia population.

Upon plotting the first two eigenvectors on the PCA plot, the three populations were stratified based on the single dimensional variation between them. The first two principal components together accounted for 20.0% of the genetic variation, revealing different populations (**Figure 2**). PCA1 differentiates POP B and C (Costa Rica) with respect to POP A (Brazil) and accounted for 11.3% of the total genetic variation. The second principal component explains 8.7% of the total variance and separated the populations from Costa Rica (POP B and C) into two different clusters. To assess the genetic diversity within populations, we calculated the observed/expected heterozygosity ratio (H_o_/H_e_). We found values of 0.23/0.34, 0.26/0.35 and 0.26/0.36 for POP A, POP B and POP C, respectively. Similar levels of genetic differentiation were found between POP A and POP BC and POP A and POP C (Fst = 0.072 ± 0.11 and Fst = 0.070 ± 0.10), whereas a lower Fst value was observed between POP B and POP C (Fst = 0.056 ± 0.09).

**Figure 3:**
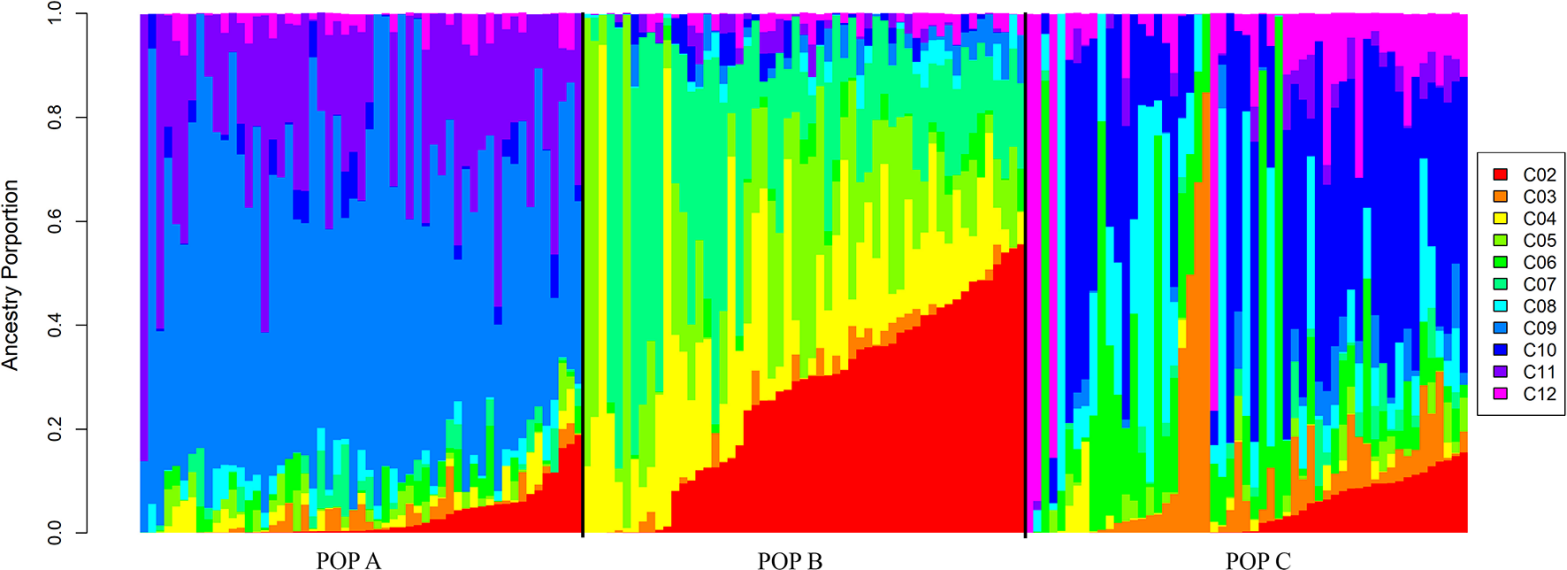
Admixture clustering of the three Nile population for K = 11. The animals are grouped by population and each individual is represented by a vertical bar. The gradient black lines delineate different populations under study and each color represent a different cluster ranged from 2 to 12 (C02 to C12).

In the admixture analysis, the posterior probability (Pr) of the fitted admixture model to the data was computed using K-values from 1 to 12 (**Supplementary Table 1**). After several runs of MCMC for each K-value (Pritchard et al., 2000), the best result was obtained with K = 11. These results indicated that the three populations share higher genome proportions with each other, indicating higher admixture level and a diverse genetic composition (**Figure 3**). STRUCTURE results evaluating K values from 2 to 12 are presented in **Supplementary File 1**, while posterior probabilities are showed in **Supplementary Table 1**.

### Estimation of Linkage Disequilibrium and Effective Population Size

**Figure 4:**
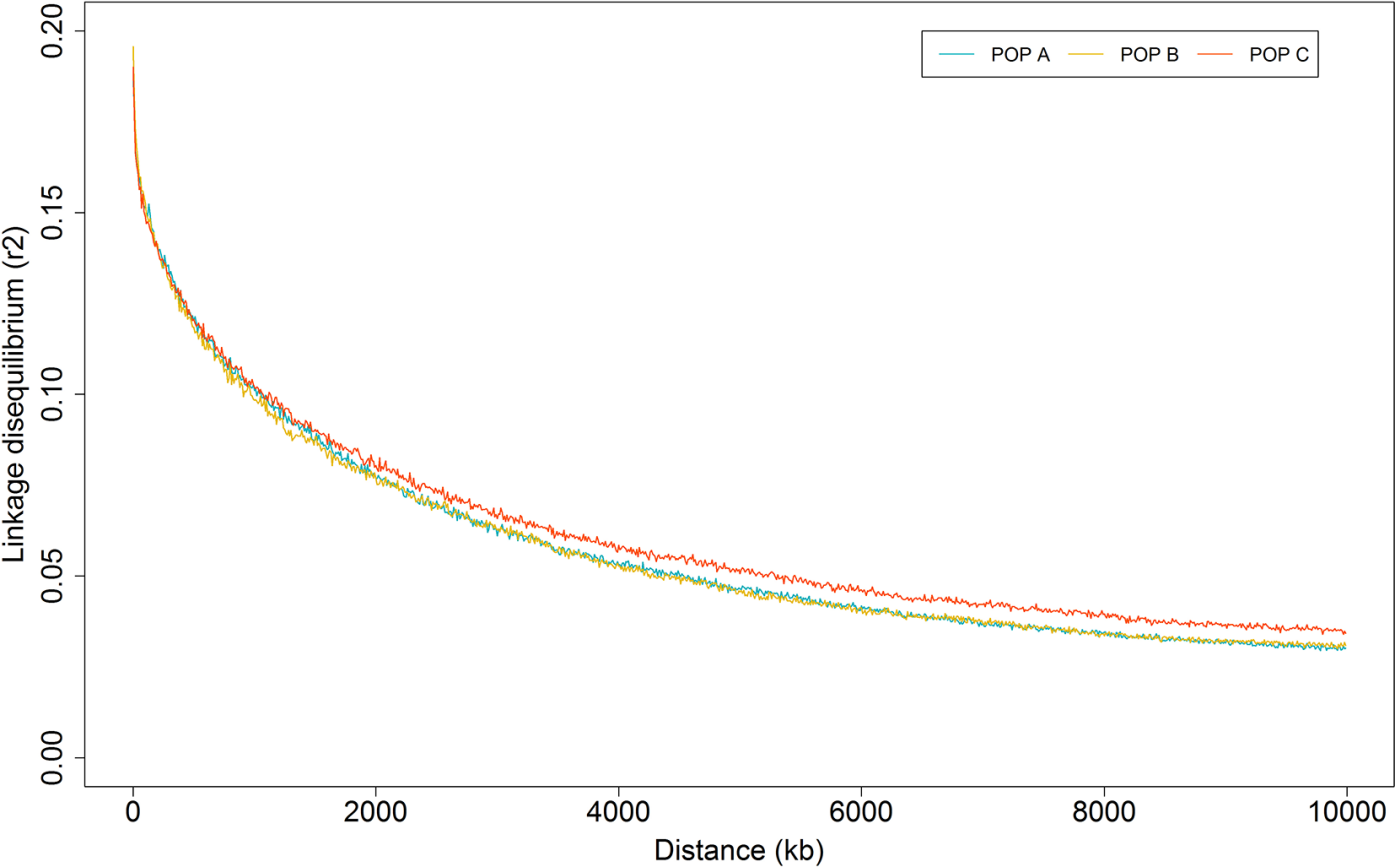
Average linkage disequilibrium decay by physical distance for three Nile tilapia population.

The overall mean LD between marker pairs measured using r^2^ was similar among populations, with values of 0.06 ± 0.10 for the three populations studied (**Table 2**). In general, the average LD among chromosomes ranged from 0.04 to 0.08 for all populations (**Table 2**). From 1 to 10,000 Kb, the average of *r* *^2^* decreased with increasing physical distance between markers, from 0.19 to 0.03 for both POP A and C, and 0.20 to 0.03 for POP B. The average LD decayed to less than 0.05 within 5 Mb (**Figure 4**), and this rate of decrease was very similar across all of the chromosomes for the three populations (**Supplementary Files 2** to **4**). In addition, the r^2^ > 0.80 were plotted for each chromosome (**Supplementary Files 5** to **7**) and suggested that for some chromosomes (e.g. LG01, LG2, LG19 and LG23) the highest r^2^ values were at both chromosome ends in the three studied populations.

**Table 2:** Number of SNPs, chromosome linkage group (LG), size in megabases (Mb), average linkage disequilibrium (r^2^) ± standard deviation (SD) and effective population size (*N* *_e_*) values for three Nile tilapia farmed populations.

Chromosome			POP A		POP B		POP C	
LG	Number of SNPs	Size (Mb)	r^2^ mean ± SD	*N* *_e_*	r^2^ mean ± SD	*N* *_e_*	r^2^ mean ± SD	*N* *_e_*
01	1,491	40.395	0.057 ± 0.092	198	0.064 ± 0.103	174	0.058 ± 0.086	137
02	1,263	36.359	0.067 ± 0.108	186	0.054 ± 0.084	190	0.065 ± 0.098	166
03	1,721	87.051	0.047 ± 0.071	230	0.050 ± 0.078	216	0.051 ± 0.076	203
04	1,295	35.337	0.049 ± 0.078	237	0.046 ± 0.073	255	0.052 ± 0.076	221
05	1,409	38.332	0.058 ± 0.094	197	0.059 ± 0.100	193	0.058 ± 0.092	200
06	1,579	42.404	0.065 ± 0.105	176	0.053 ± 0.086	224	0.060 ± 0.090	192
07	2,830	64.666	0.072 ± 0.106	141	0.068 ± 0.105	154	0.081 ± 0.116	127
08	1,300	30.391	0.059 ± 0.099	195	0.053 ± 0.086	227	0.056 ± 0.086	210
09	1,139	35.346	0.051 ± 0.081	229	0.053 ± 0.087	218	0.057 ± 0.089	197
10	1,353	34.128	0.055 ± 0.087	212	0.060 ± 0.096	195	0.065 ± 0.096	167
11	1,447	38.280	0.057 ± 0.089	203	0.054 ± 0.090	217	0.068 ± 0.098	164
12	1,607	38.490	0.052 ± 0.084	222	0.057 ± 0.094	204	0.055 ± 0.085	211
13	1,313	34.470	0.077 ± 0.137	146	0.061 ± 0.107	191	0.071 ± 0.112	155
14	1,686	39.722	0.052 ± 0.085	231	0.063 ± 0.100	179	0.072 ± 0.106	149
15	1,313	36.093	0.060 ± 0.091	189	0.065 ± 0.102	179	0.068 ± 0.105	167
16	1,594	35.701	0.065 ± 0.102	184	0.066 ± 0.104	191	0.062 ± 0.098	195
17	1,443	38.625	0.063 ± 0.093	182	0.059 ± 0.093	205	0.055 ± 0.083	219
18	1,437	38.551	0.062 ± 0.103	186	0.058 ± 0.100	209	0.062 ± 0.099	184
19	1,254	30.915	0.074 ± 0.137	159	0.089 ± 0.155	128	0.076 ± 0.123	158
20	1,519	36.847	0.057 ± 0.089	201	0.056 ± 0.089	212	0.054 ± 0.083	215
22	1,624	38.436	0.054 ± 0.085	206	0.055 ± 0.092	208	0.068 ± 0.110	163
23	1,619	45.291	0.056 ± 0.096	210	0.056 ± 0.093	210	0.066 ± 0.105	179
**Average**	**1,511**	**40.720**	**0.060 ± 0.096**	**196**	**0.059 ± 0.096**	**199**	**0.063 ± 0.098**	**181**

**Figure 5:**
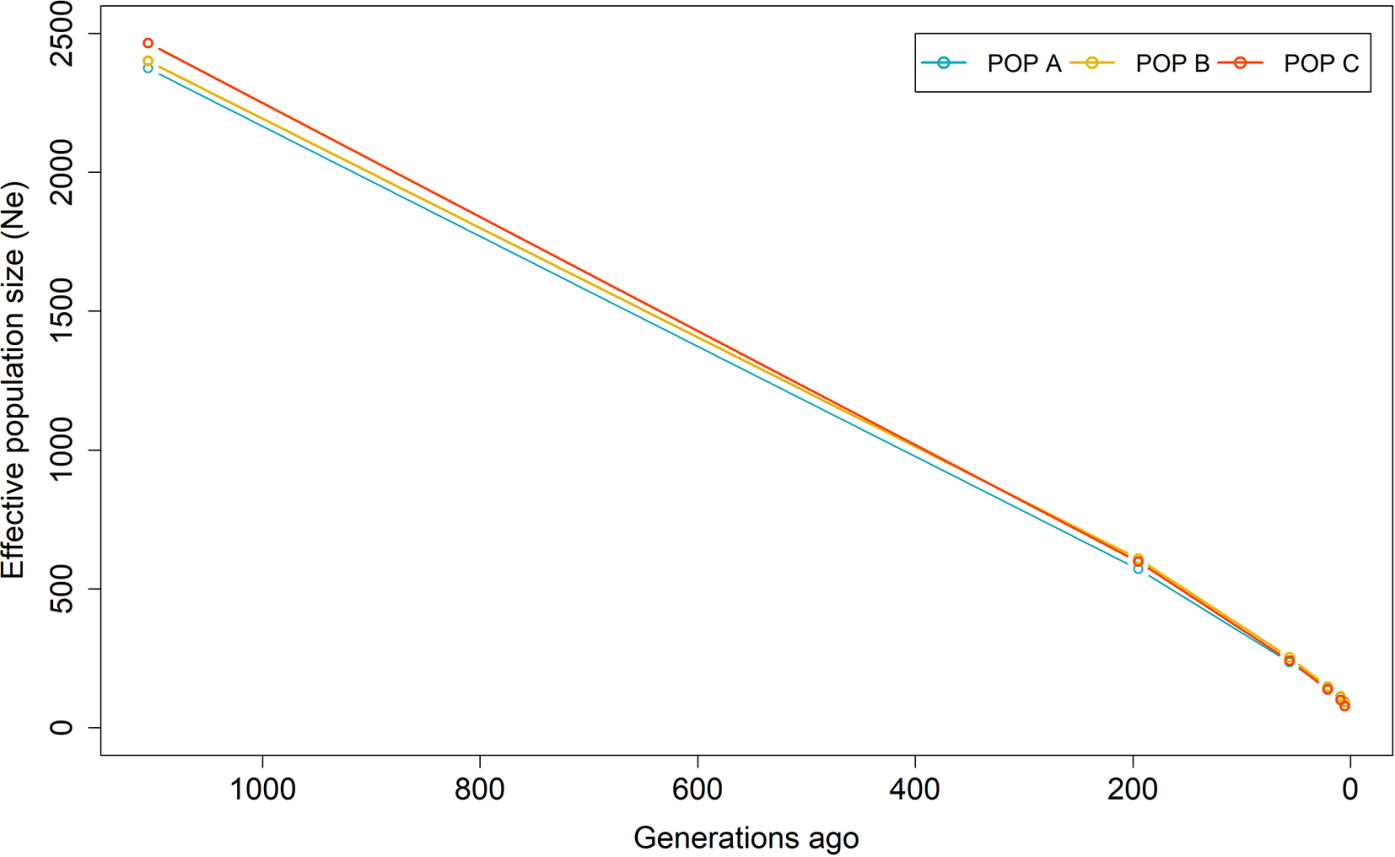
Effective population size (*N* *_e_*) from 1,105 to 5 generations ago based on linkage disequilibrium for three Nile tilapia populations.

**Figure 5** shows the historical *N*_e_ from 1,105 to 5 generations ago. The *N* *_e_* values were lower in the recent past than the distant past. These values calculated at five generations ago were 93, 90 and 78 for POP A, POP B and POP C, respectively. The harmonic means for *N* *_e_* at five to 1,105 generations ago was 196, 199 and 181 for POP A, POP B and POP C, respectively. In addition, the *N* *_e_* varied among chromosomes, ranging from 127 to 255 (**Table 2**). Recent *N* *_e_* calculated based on LD values were 159, 128 and 78 for POP A, POP B and POP C, respectively, whereas the regression on historical *N* *_e_* resulted in contemporary *N* *_e_* values of 111, 121 and 106 for POP A, B, and C respectively. Based on the effective number of chromosome segments, a minimum number of markers for a high power genomic analysis should be at least 9,400, 7,600, and 4,600 for POP A, POP B, and POP C, respectively.

## Discussion

### Genomic Population Structure

In the PCA, the first two principal components explained about 20% of the total genetic variation for the populations studied and clearly revealed three different clusters, corresponding to the three populations present in the dataset (**Figure 2**). In addition, the low value of H_o_ in relation of H_e_ suggest a loss of genetic diversity due to founder effect or effective population size.

The admixture results provided evidence of a recent mixture of different strains to conform highly admixture populations. Although the PCA demonstrates three distinct populations, the admixture analysis suggested that, the three Nile tilapia populations studied are related through the common GIFT origin. The genetic differentiation among populations may have been partly generated by genetic drift or founder effect events which can have a pronounced effect on allele frequencies (Allendorf and Phelps, 1980). Furthermore, the three populations have undergone artificial selection for the improvement of growth-related traits in different geographic locations, exposing the populations to distinct environmental conditions and production systems. This could be observed especially in the comparison between the population from Brazil and from Costa Rica. POP A from Brazil is evaluated in cage-based production system during the autumn and winter season, whereas both POP B and C, from Costa Rica, are evaluated in pond-based conditions, during winter and spring season. Furthermore, environmental conditions as temperature and rainfall are different between the countries. In Brazil, the temperature ranges from 10 to 29°C during the year and the rainfall period coincides with the spring and summer season, which is different in Costa Rica, where the wet periods coincide with autumn and winter, and the temperature is rarely lower than 22°C.

### Linkage Disequilibrium and Effective Population Size

Evaluating the whole-genome LD within populations, may help to understand the different demographic processes experienced by these populations. These processes include admixture, mutation, founder effect, inbreeding and selection (Gaut and Long, 2003). This is the first study aimed at estimating the extent and decay of LD in farmed Nile tilapia populations established in Latin America (specifically, Brazil and Costa Rica), and artificially selected for growth-related traits. Measures to reduce biasness included the removal of animals with high IBD, as described in methodology. Thus, we used a similar number of animals from each population. Similarly, alleles with high frequency result in less biases estimation of LD (Espigolan et al., 2013). In the present study a small proportion of SNP (<13%) have MAF lower than 0.10 and low IBD values indicating an accurate estimation of LD.

Accurate LD estimations depend on the different factors including sample size and relatedness among individuals. In the current work we used 55 animals for each population, as it has been suggested by Bohmanova et al. (2010) and Khatkar et al. (2008). Furthermore, we used r^2^ as a measure of LD instead of |D’| to avoid the likely overestimation of LD due to this sample size (Khatkar et al., 2008).

We updated the order and positions of the SNP on the 50K SNPs from Illumina BeadChip panel (Yáñez et al., 2019) to the most recent Nile tilapia genome reference (O_niloticus_UMD_NMBU, Conte et al., 2019), to get a more accurate intermarker distances. However, we observed on chromosome LG13 and LG19, a pool of *r* *^2^* values > 0.40 for pair-wise SNP at large distances (>7 Mb; **Supplementary Files 2** to **4**), but a decline in LD with the increase in physical distance between markers is expected. Incorrect position of SNPs on the reference genome or errors in the reference genome assembly might have resulted in these errors. Our study revealed that the LD level declined to 0.05 at the inter-marker distance of 5 Mb and that the decay patterns were similar between populations (**Figure 4**). A previous study conducted by Hong Xia et al. (2015) reported similar LD patterns for GIFT tilapia stocks collected from South Africa, Singapore and China. Using microsatellite, Sukmanomon et al. (2012) estimated LD means in terms of the disequilibrium coefficient (D’) of 0.05 for a GIFT population originating from the Philippines. Whereas, Conte et al. (2019) and Joshi et al. (2018) using a dense marker panel (>40 K) reported higher LD values at the end of LGs and low values at the middle, supported by the identification of a sigmoidal pattern of recombination in most of the chromosomes, with high and low recombination rates at the middle and both chromosome extremes, respectively. We found similar LD patterns in some chromosomes for the three farmed Nile tilapia populations from Latin America studied here, nevertheless we found a smaller number of marker pairs that are in high LD, compared to Conte et al. (2019).

Due to differences between genomes, the quality control applied and population structure, LD comparison between species is inappropriate, however we used references from other farmed fish species because of the limited information that exists for this kind of study in tilapia. The tilapia population seems to present a weaker short-range LD than other farmed fish populations (Gutierrez et al., 2015; Kijas et al., 2016; Barria et al., 2018a; Barria et al., 2018c; Vallejo et al., 2018). A likely explanation is due the diverse origin of the base population used to form the Nile tilapia populations studied here, as it has also been suggested for a Chilean farmed Atlantic salmon population with Norwegian origin (Barria et al. 2018c). In salmonids, some suggest admixture is a major factor contributing to long-range LD (Ødegård et al., 2014; Barria et al., 2018c; Vallejo et al., 2018). Our results suggest that there is evidence of recent admixture in the three studied populations with introgression of multiple strains with different origins. However, this admixture process has not resulted in long-range LD, suggesting that other biological and demographic processes are also important in the current levels of LD in POP A, B and C, including recombination rates and effective population size.

Linkage disequilibrium at a short distance is a function of effective population size many generations ago and LD at long distances reflect the recent population history. The LD estimation at small and large distance, have similar pattern for the three Nile tilapia populations (**Figure 4**). These results reflected in slight difference in *N* *_e_* value of many generations ago and in the recent past among populations (**Figure 5**). However, the continuous reduction in the *N* *_e_*, was observed over the previous 1,105 generations (**Figure 5**). The three populations in this study have been under artificial selection for several generations. The reduction of *N* *_e_* can be an indicator of selection and suggested an important cause of increased LD (Pritchard and Przeworski, 2001). The use of a common GIFT strain as genetic basis to form the POP A, B, and C and similar demographic processes among them (recent admixture and selection), may have resulted in the similar pattern of LD and historical *N* *_e_*. Among the chromosomes, the highest LD mean value (ranging from 0.04 to 0.09) and also the lowest effective population size (<161) was reported for LG7, LG13 and LG19 (**Table 2**). The variation in autosomal recombination rates among chromosomes (Conte et al., 2019) leads to diversity in the pattern of LD in different genomic regions. In addition, differences in the LD can be attributed to the number of markers analyzed among chromosomes, their MAF values and also the effect of artificial selection across the genome (López et al., 2015).

The contemporary *N* *_e_* estimated using both the NeEstimator v2.01 (Do et al., 2014) software and the regression of historical *N* *_e_* values, resulted in the same *N* *_e_* expanded pattern. The most likely explanation for the increasing *N* *_e_* in the recent generations is because of the recent establishment of these composite populations based on the hybridization of different Nile tilapia strains 5 to 10 generations back (Cáceres et al., 2019). Moreover, the selection and mating methods for these populations are based on the optimization of the contributions from parents to progeny; minimizing the average co-ancestry among progeny, reducing the inbreeding level (Meuwissen, 1997; Kinghorn, 1998), and maximizing the effective population size (Caballero and Toro, 2000). Previously, similar value of *N* *_e_* was estimated using pedigree information from a GIFT population from Malaysia (Ne = 88) (Ponzoni et al., 2010). Some authors suggest keeping *N* *_e_* values between 50 and 200 to ensure genetic variability and diversity in a long-term breeding population (Smitherman and Tave, 1987). In contrast to the results found here, a smaller *N* *_e_* was found for farmed rainbow trout (Vallejo et al., 2018) and Atlantic salmon with North American and European origins (Kijas et al., 2016; Barria et al., 2018c).

In summary, within tilapia populations, the LD values were very low even in short distances (*r* *^2^* = 0.15 for markers spaced at 20–80 Kb). Similar values were found in humans (Reich et al., 2001; Ardlie et al., 2002), coho salmon (Barria et al., 2018a), some breeds of cattle (de Roos et al., 2008; Khatkar et al., 2008; Yurchenko et al., 2018), sheep (Alvarenga et al., 2018) and goats (Brito et al., 2015).

### Practical Implications

The LD results have several implications for future implementation of genomic tools in the current farmed Nile tilapia populations. Both GWAS and genomic selection are dependent on LD extent to define the number of SNPs necessary to assure the causative mutation variance (Flint-Garcia et al., 2003) and to achieve a certain accuracy of genomic estimated breeding value (Meuwissen et al., 2001). Meuwissen (2009) suggested that to achieve accuracies of genomic breeding (GEBV) ranging from 0.88 to 0.93 using unrelated individuals; it is necessary to have *2N* *_e_* *L* number of individuals and *10N* *_e_* *L* number of markers, where L is the length of genome in Morgans. In our study, the contemporary *N*_e_ is 159, 128 and 78 for POP A, POP B and POP C, respectively, and the length of the genome is 14.8 Morgans (Joshi et al., 2018; Conte et al., 2019). Thus, the 11,500 to 23,500 markers will be required for unrelated Nile tilapia populations. In contrast, Goddard (2009) suggested that accuracy of genomic prediction is highly dependent on the effective number of chromosome segments (*M* *_e_* = *4N* *_e_* *L*).

Having a number of independent, biallelic and additive QTL affecting the trait we would need a smaller number of markers to achieve a high accuracy. Thus, the minimum number of markers for a high-power genomic analysis should be at least, 9,400, 7,600, and 4,600 for POP A, POP B, and POP C, respectively. Despite the fact that these numbers were slightly lower than those suggested by Vallejo et al. (2018) and Barria et al. (2018a) for rainbow trout and coho salmon, respectively, alternative methods are necessary for cost-efficient genomic application in tilapia breeding programs.

A recent study tested different marker densities and imputed genotypes to assess genomic prediction accuracies in a farmed Nile tilapia population. The prediction accuracy using genomic information outperformed the estimated breeding values using the classical pedigree-based best linear unbiased prediction, even using a very low-density panel (0.5K) for growth and fillet yield (Yoshida et al., 2019b). In addition, the high values of imputation accuracy (>0.90) were not affected by the linkage disequilibrium pattern, probably due to the family-based population structure and high relatedness among animals, suggesting that genomic information may be cost-effectively included in Nile tilapia breeding programs.

## Conclusions

The current study revealed similar short-range LD decay for three farmed Nile tilapia populations. The PCA suggested three distinct populations and the admixture analysis confirmed that these three populations are highly admixed. Based on the number of independent chromosome segments, at least 9.4, 7.6, and 4.6 K SNPs for POP A, B, and C, respectively might be required to implement genomic prediction in the current Nile tilapia populations, whereas for GWAs studies more markers may be necessary to achieve higher power and greater precision for QTL detection.

## Ethics Statement

The sampling protocol was previously approved by The Comité de Bioética Animal, Facultad de Ciencias Veterinarias y Pecuarias, Universidad de Chile (certificate N° 18179-VET-UCH).

## Author Contributions

GY performed the analysis and wrote the initial version of the manuscript. AB contributed with discussion and writing. GC, MC and AJ performed DNA extraction. KC and JL contributed with study design. JY conceived and designed the study; contributed to the analysis, discussion and writing. All authors have reviewed and approved the manuscript.

## Funding

This work has been funded by Corfo (project number 14EIAT-28667).

## Conflict of Interest Statement

GY, JPL and KC were hired by a commercial institution (Benchmark Genetics Chile) during the period of the study. The remaining authors declare that the research was conducted in the absence of any commercial or financial relationships that could be construed as a potential conflict of interest.
