# Supplementary figures and images for: Genome-Wide Patterns of Population Structure and Linkage Disequilibrium in Farmed Nile Tilapia (Oreochromis niloticus)
Source: Front Genet. 2019 Sep 4;10:745. doi: 10.3389/fgene.2019.00745 (PMC6737105; doi:10.3389/fgene.2019.00745)

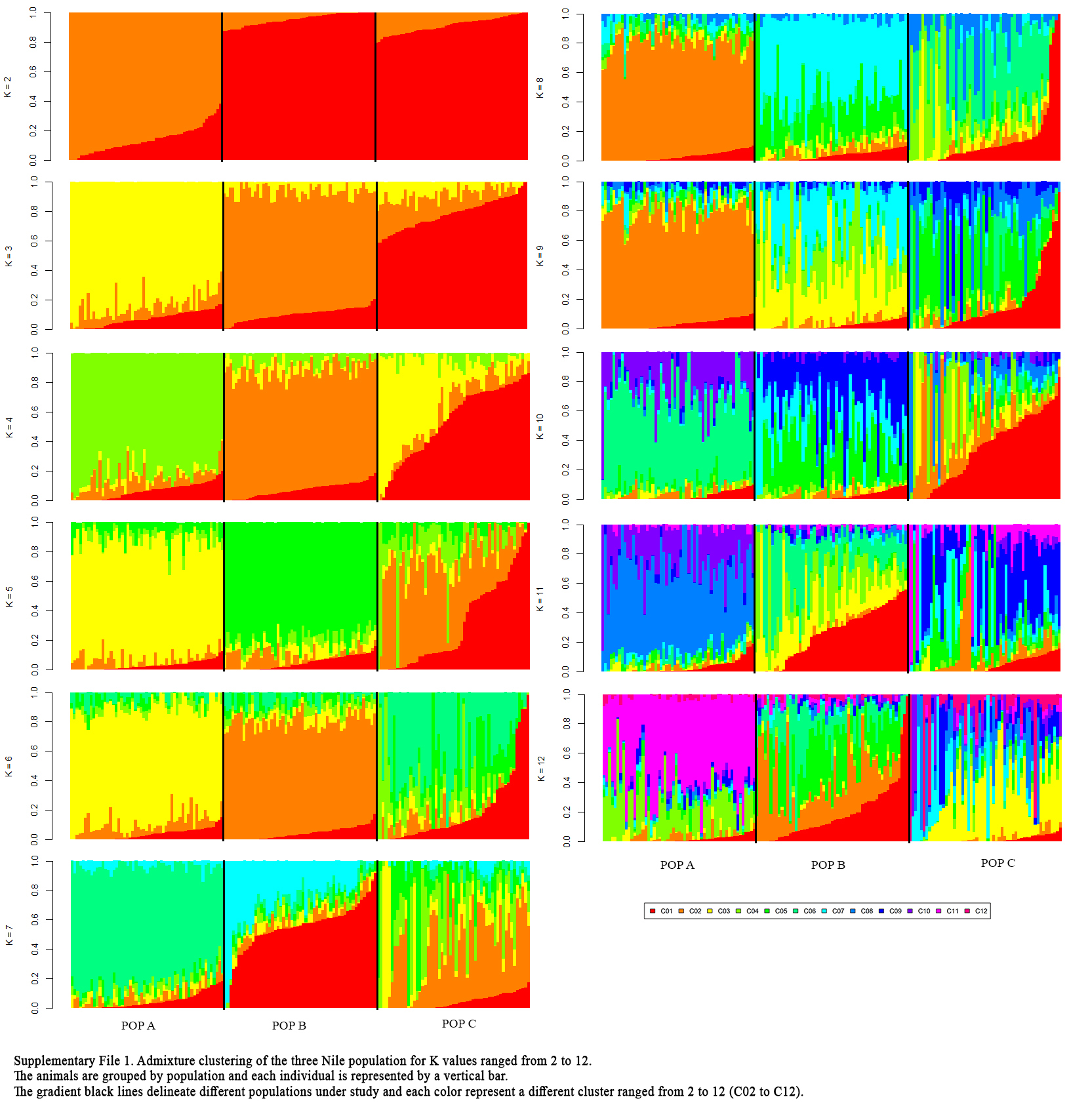

Supplement: Supplementary file 1 [file Image_1.jpg]

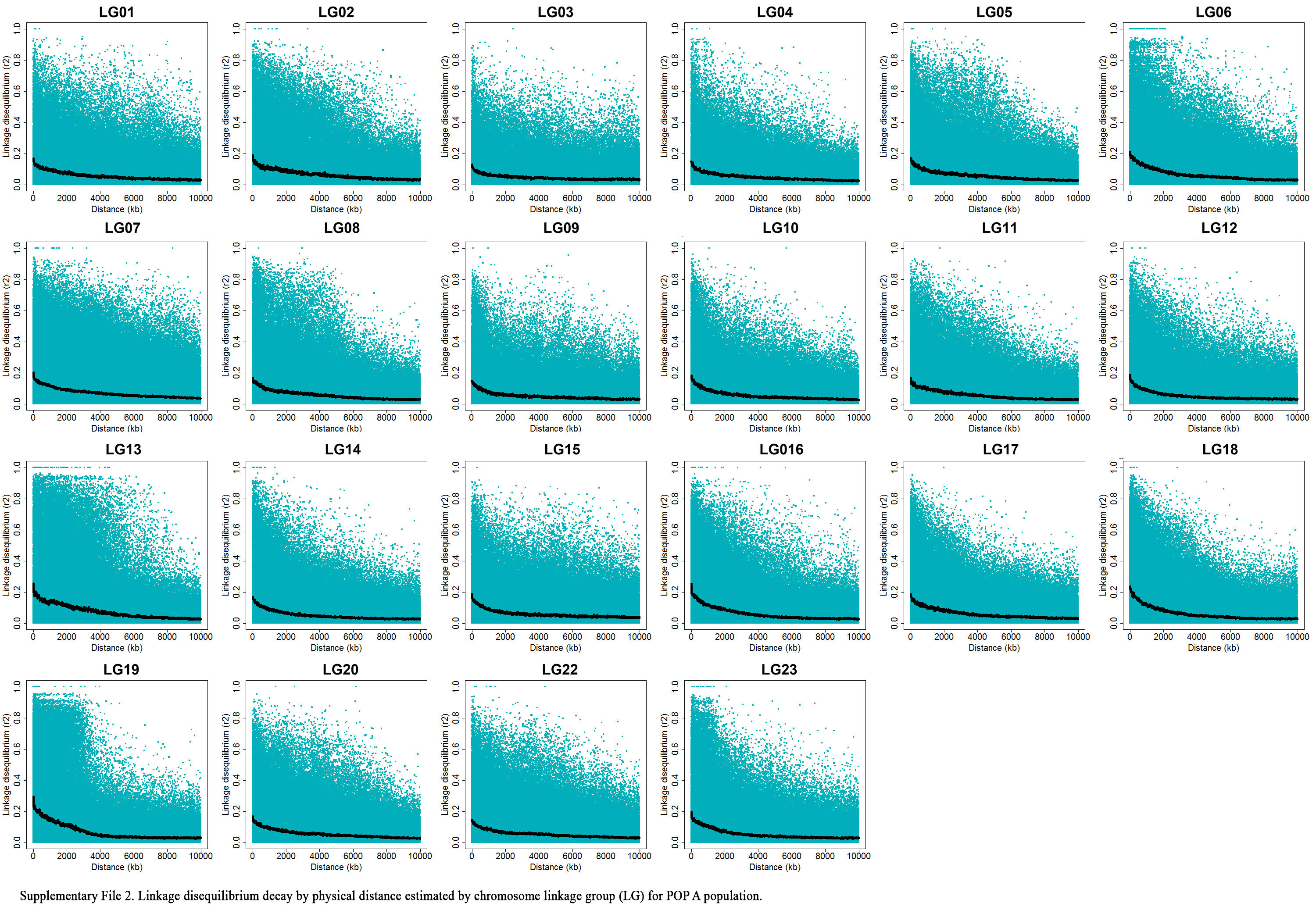

Supplement: Supplementary file 2 [file Image_2.jpg]

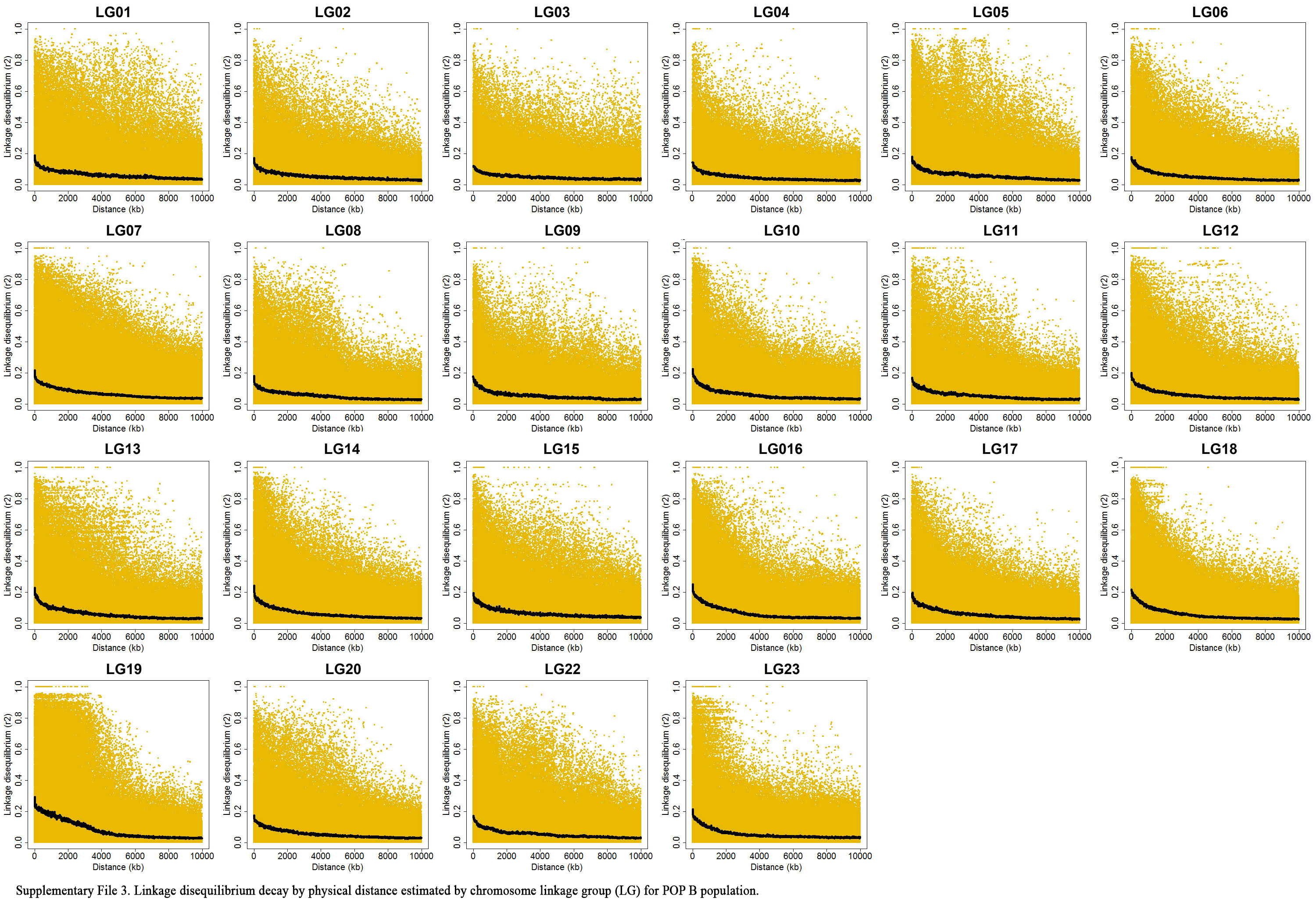

Supplement: Supplementary file 3 [file Image_3.jpg]

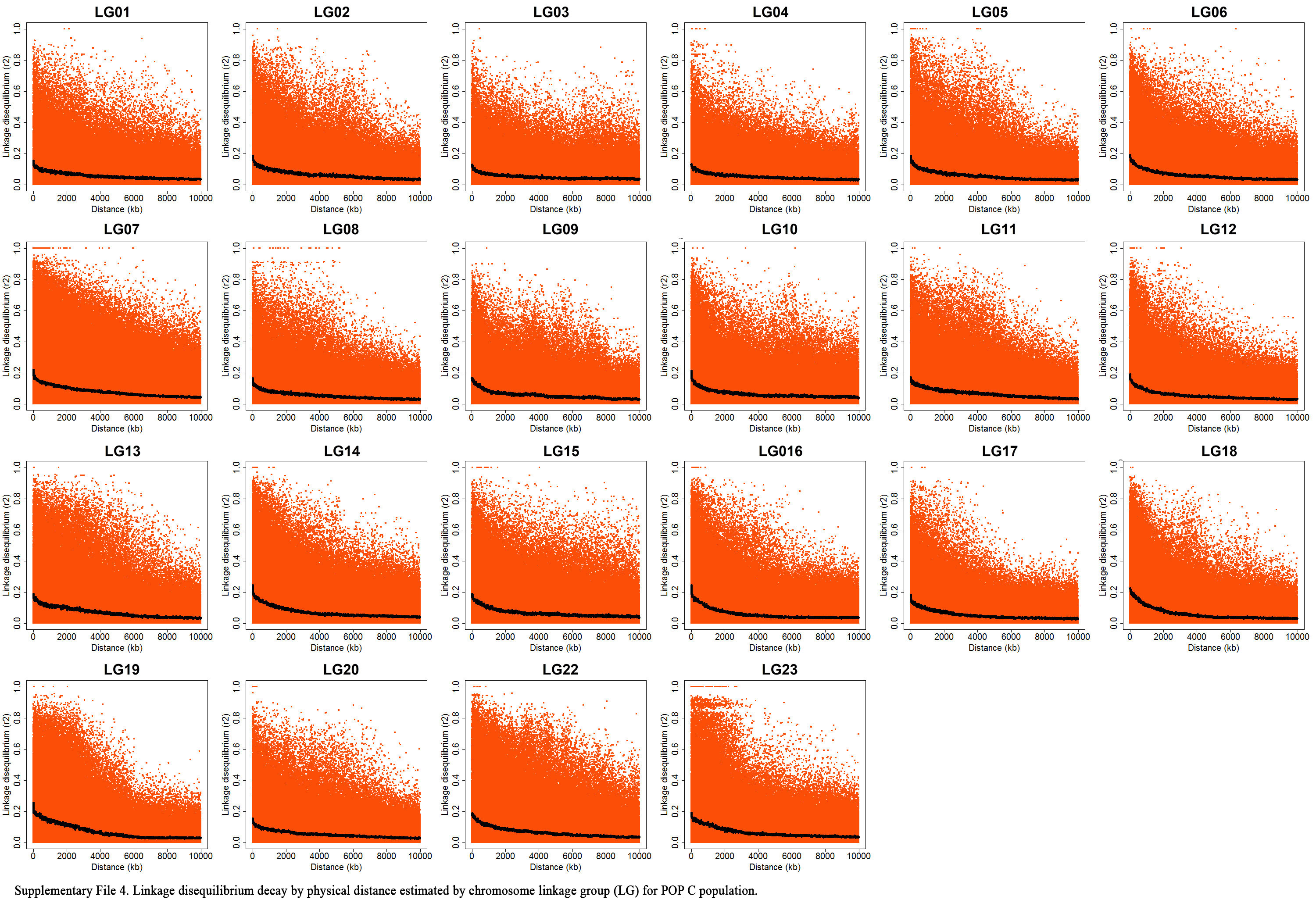

Supplement: Supplementary file 4 [file Image_4.jpg]
